# High‐Protein Diet Exacerbates Insulin Resistance via the JNK/IKKβ‐IRS‐1 Pathway

**DOI:** 10.1002/edm2.70147

**Published:** 2025-12-12

**Authors:** Junjun Li, Jinhua Xu, Jinmiao Tian, Lixia Chen, Lili Zhao, Yongming Yang, Jing Wang, Lei Yan, Yuxin Yang, Yuchen Jiang, Simin Chen, Binxuan Wang, Lulu Wang, Xihua Yang

**Affiliations:** ^1^ Shanxi Medical University Affiliated Cancer Hospital Taiyuan China; ^2^ Shanxi Medical University School of Public Health Taiyuan China; ^3^ Laboratory Animal Center Shanxi Province Cancer Hospital Taiyuan China

**Keywords:** high‐protein diet, insulin resistance, type 2 diabetes

## Abstract

**Objective:**

To investigate the effects of a high‐protein (HP) diet on insulin resistance in a type 2 diabetes (T2D) mouse model.

**Methods:**

A high‐fat diet combined with streptozotocin (STZ, 25 mg/kg) was used to induce a T2D model. Fasting blood glucose levels > 7 mmol/L were considered successful modelling. The models were further divided into three groups: the Normal‐Protein‐T2D (TN, *n* = 8), High‐Protein‐T2D (TH, *n* = 8), and High‐Fat‐T2D (TF, *n* = 8) groups. These groups were fed diets containing 20% protein, 60% protein, and 60% fat, respectively. Additionally, 24 Kunming (KM) mice, serving as non‐T2D controls, were divided into a Normal‐Protein‐Control (N, *n* = 8), a High‐Protein‐Control (H, *n* = 8), and a Low‐Protein‐Control (L, *n* = 8), fed 20% protein, 60% protein, and 5% protein diets, respectively. After 6 weeks of dietary intervention, FBG, serum biochemical markers, hepatic inflammatory factors and pathological changes, and pancreatic immunohistochemistry were assessed. Expression of IRS‐1, JNK, IKKβ, and their phosphorylated proteins were analysed.

**Results:**

Mice in the H group exhibited significantly impaired glucose tolerance, exacerbated insulin resistance, and reduced liver function. In the T2D model, TH mice exhibited more severe hyperglycemia, insulin resistance, and β‐cell dysfunction than TN mice, accompanied by increased hepatic TNF‐α and IL‐6 expression, enhanced lipid accumulation, suppressed IRS‐1 phosphorylation, and enhanced JNK and IKKβ phosphorylation.

**Conclusion:**

A HP diet induces insulin resistance in normal mice and further exacerbates glucose metabolism disorders in T2D models, suggesting that restricting HP intake may serve as a strategy for preventing T2D.

## Graphical abstract

A high‐protein diet worsens insulin resistance and β‐cell dysfunction in both normal and T2D mice by increasing hepatic inflammation and suppressing IRS‐1 signalling. Limiting excessive protein intake may help prevent or delay the progression of type 2 diabetes.

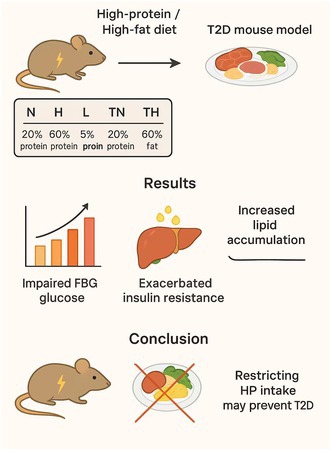

## 1 Introduction

Protein plays a vital role in human nutrition, providing amino acids essential for muscle development and acting as regulatory molecules in vital organs and immune cells [1]. High‐protein diets (HP) are rich in methionine and branched‐chain amino acids, improving body composition and energy balance [2], significantly reducing hepatic fat [3], lowering blood glucose levels, and enhancing insulin sensitivity [4]. However, excessive protein intake may induce insulin resistance in non‐diabetic individuals [1], complicate glucose homeostasis [5], exacerbate insulin resistance, promote gluconeogenesis [6] intensify pro‐aging effects triggered by compensatory hyperinsulinemia [7] and potentially increase the risk of metabolic disorders such as Type 2 Diabetes (T2D).

Although the pathogenesis of T2DM remains incompletely understood, it likely involves inflammation and insulin resistance [8]. Zhang et al. [9] found that the mechanism linking dietary protein to insulin resistance likely involves inflammatory signalling pathways, similar to high‐fat diets [10]. Both JNK (c‐Jun N‐terminal kinase) and IKKβ (IκB kinase) pathways, activated by inflammatory cytokines, participate in diet‐induced insulin resistance [9, 11]. IKKβ not only phosphorylates IKBα and activates NF‐κB but also phosphorylates insulin receptor substrates (IRS), thereby inhibiting insulin signalling [12]. As a core organ for insulin signalling and glucose/lipid metabolism regulation [13], the liver is particularly susceptible to insulin resistance mediated by IRS‐1 post‐translational modifications [14]. Although numerous studies have explored how excessive protein intake may induce insulin resistance and T2D, the effects of chronic HP intake on insulin sensitivity and blood glucose levels in healthy populations remain unclear [1]; moreover, its role in glucose homeostasis remains undefined [15].

Therefore, this study employs a T2D mouse model to systematically monitor glycemic dynamics and insulin resistance markers, aiming to elucidate the regulatory effects of HP diets on insulin resistance and clarify its underlying molecular mechanisms. This research seeks to provide robust experimental evidence for the scientific application of HP diets in clinical nutritional interventions for T2D.

## 2 Materials and Methods

### 2.1 Experimental Animals and Study Design

SPF‐grade Kunming (KM) mice (male, *n* = 48, initial weight 12 ± 3 g) were purchased from the Experimental Animal Center of Shanxi Cancer Hospital [Animal Production Licence No.: SYXK(Jin)2022‐0002, Use Licence No.: SYXK(Jin)2022‐0003]. Forty‐eight Kunming mice were randomly divided into four groups: the Normal‐Protein‐Control (N, *n* = 8, 20% protein), High‐Protein‐Control (H, *n* = 8, 60% protein) [16], Low‐Protein‐Control (L, *n* = 8, 5% protein), and T2D model (*n* = 24, 60% high‐fat) [17] groups. For clarity and convenience in figures, tables, and text, these groups are referred to by the abbreviations N, H, and L throughout the manuscript. After four weeks of diet‐controlled feeding, an oral glucose tolerance test (OGTT) was performed on the N, H, and L groups, followed by an insulin tolerance test (ITT) one week later. After one month of high‐fat diet [18], the T2D group was induced with streptozotocin (STZ, 25 mg/kg) via intraperitoneal injection to develop diabetes (defined as fasting blood glucose > 7 mmol/L) [19]. The group was further divided into Normal‐Protein‐T2D (TN, 20% protein), High‐Protein‐T2D (TH, 60% protein), and High‐Fat‐T2D (TF, 60% fat). For clarity and convenience in figures and text, these groups are referred to by the abbreviations TN, TH, and TF throughout the manuscript. The experiment lasted 16 weeks with ad libitum feeding. Body weight was recorded every 10 days and blood glucose measured every 2 weeks. An oral glucose tolerance test (OGTT) was performed at week 4 post‐diabetes induction, and the experiment concluded at week 6. All animals were euthanized after a 12‐h fast. Prior to euthanasia, mice were anaesthetised via intraperitoneal injection of tribromoethanol (0.2 mL/10 g body weight, Nanjing Aibei Biotechnology Co. Ltd.). Blood samples were collected via retroorbital puncture, allowed to stand at 4°C for 30 min, then centrifuged at 3000× *g* for 10 min to separate serum. Serum samples were analysed for aspartate aminotransferase (AST), alanine aminotransferase (ALT), total cholesterol (TC), triglycerides (TG), high‐density lipoprotein (HDL), low‐density lipoprotein (LDL), glucose (GLU) levels. Enzyme‐linked immunosorbent assay (ELISA) was used to measure insulin (INS), mouse tumour necrosis factor‐alpha (TNF‐α), and interleukin‐6 (IL‐6). A portion of liver tissue was fixed in 4% paraformaldehyde for subsequent haematoxylin–eosin (HE) staining and Oil Red O staining. Another portion was cryopreserved at −80°C for Western Blot (WB) analysis. Pancreatic tissue fixed with 4% paraformaldehyde was used for insulin and glucagon immunohistochemistry (IHC) staining. All experimental protocols were approved by the Shanxi Cancer Hospital Animal Welfare and Ethics Review Committee (Ethics Approval No.: 2024017).

### 2.2 Animal Feed

Customised feed provided by Xiaoshuyoutai Servicebio Technology Co. Ltd. [Production Licence No.: SCXK (Beijing) 2018‐0006]. All feeds are calorie‐equivalent but differ in macronutrient composition. The high‐protein diet contains 60% protein, 25% carbohydrates, and 16% fat (percent of total calories), fed to Groups H and TH. The normal‐protein diet contained 20% protein and was fed to Groups N and TN. Except for the high‐fat diet, fat calories in all other diets were uniformly controlled at 16%. The high‐fat diet derived 60% of its energy from fat and was fed to Group TF. Specific macronutrient ratios and feeding groups are detailed in Table 1.

**TABLE 1:** Dietary macronutrient composition ratios.

Feed	Protein		Carbohydrates		Fat		Feed calories
	g %	kcal %	g %	kcal %	g %	kcal %	(kcal/g)
Group H, TH	60.3	60.91	22.9	25	7	16	3.96
Group N, TN	20.3	20.51	62.9	65	7	16	3.96
Group L	5.3	5.35	77.9	80	7	16	3.96
Group TF	26.2	20	26.3	20	34.9	60	3.96

### 2.3 OGTT and ITT

Four weeks after successful induction of the T2D model, mice were administered a glucose solution via gavage at 0.1 mL/10 g body weight. Following an 8‐h fast (with free access to water), baseline blood glucose levels were measured in the tail vein (0 min). Glucose levels were monitored at 30, 60, 90, and 120 min after the glucose challenge using the Accu‐Chek blood glucose meter (Roche Diagnostics, Germany). One week later, mice underwent ITT after 4‐h fasting. A 4% glucose solution was prepared for emergency use. Insulin was administered via intraperitoneal injection at 0.75 U/kg, and blood glucose was measured at the same time points. Using the baseline 0 min value as reference, the area under the glucose response curve (AOC) was calculated using the trapezoidal method.

### 2.4 Serum Biochemical Analysis and ELISA

Serum biochemical parameters (AST, ALT, TC, TG, HDL, LDL, GLU) were measured using the Mindray BS‐240VET fully automated biochemical analyser (Shenzhen Mindray Bio‐Medical Electronics Co. Ltd.). INS, TNF‐α, and IL‐6 were detected using ELISA kits (Beijing Andy Huatai Technology Co. Ltd). Serum samples were stored at −20°C until testing, thawed at room temperature, thoroughly mixed, and tested in duplicate according to manufacturer protocols. Separately, liver tissue stored at −80°C was thawed at 4°C and homogenised in pre‐chilled PBS. After centrifugation at 3000× *g* for 10 min at 4°C, the supernatant was collected for detection of TNF‐α and IL‐6. All assays were read at 450 nm wavelength. Concentrations were calculated using standard curves, and the mean values were used for subsequent statistical analysis.

### 2.5 HE and Oil Red O Staining

Liver tissue was rinsed with physiological saline to remove blood. A portion of the tissue was stained with Oil Red O to assess lipid accumulation. The remaining tissue was fixed with 4% paraformaldehyde, embedded in paraffin, sectioned, and subjected to HE staining. Histopathological evaluation was performed by photographing images under an optical microscope. Lipid droplet area was calculated using Image J software.

### 2.6 Immunohistochemistry

Pancreatic tissue was fixed overnight at 4°C in 4% formaldehyde, paraffin‐embedded, and sectioned (5 μm). Sections were incubated at room temperature in 3% hydrogen peroxide for 25 min to block endogenous peroxidase activity. Primary antibodies against insulin and glucagon (1:5000, Wuhan Saiwei Biotechnology) were at 1:5000 dilution and incubated overnight at 4°C. After washing, secondary antibody (1:5000; Wuhan Servicebio Technology Co. Ltd) was at 37°C for 1 h. After incubation, sections underwent immunostaining and TSA counterstaining. Visualisation was performed using CaseViewer 2.4, and pancreatic area along with α‐ and β‐cell areas were quantified using Image J software.

### 2.7 WB

Protein levels of IRS‐1, JNK, IKKβ, and their phosphorylated forms were detected in liver tissue via WB. Liver tissue was homogenised in Radio‐Immunoprecipitation Assay Lysis Buffer (RIPA) containing 1% protease inhibitor (Wuhan Servicebio Technology Co. Ltd) and 1% phosphatase inhibitor (Shanghai Epizyme Biomedical Technology Co. Ltd). The homogenate was centrifuged at 12,000× *g* for 15 min at 4°C, and the supernatant was collected. Protein concentration was determined using the BCA method, and all samples were adjusted to a uniform concentration. After denaturing the protein samples, SDS‐PAGE electrophoresis was performed (80 V for 15 min, 120 V for 60 min), followed by transfer to a PVDF membrane. The membrane was incubated overnight at 4°C with the specific primary antibody (Affinity Biosciences Co. Ltd., diluted 1:1000). After washing, it was incubated at room temperature for 1 h with HRP‐labelled secondary antibody (HRP‐labelled goat anti‐mouse IgG secondary antibody, diluted 1:5000). Finally, the membrane was developed using the Omni‐ECL chemiluminescent detection kit (Epizyme Bio. Co. Ltd., Shanghai, China), and band intensities were analysed using ImageJ software.

### 2.8 Statistical Analysis

Data underwent Shapiro–Wilk normality testing and Levene's test for homogeneity of variance. For normally distributed data with homogeneous variance, one‐way ANOVA was performed, followed by Bonferroni post hoc comparisons; for heterogeneous variance, Dunnett's T3 test was used. Non‐normally distributed data underwent Kruskal–Wallis test followed by Dunn's multiple comparisons. Results are presented as mean ± standard error of the mean (Mean ± SEM). Statistical analysis was performed using SPSS 27 software, with figures generated by GraphPrism 8.0.2. *p* < 0.05 was considered statistically significant, denoted by ‘abc’.

## 3 Results

### 3.1 HP Diet Induces Insulin Resistance and Impaired Glucose Tolerance in Mice

**FIGURE 1:**
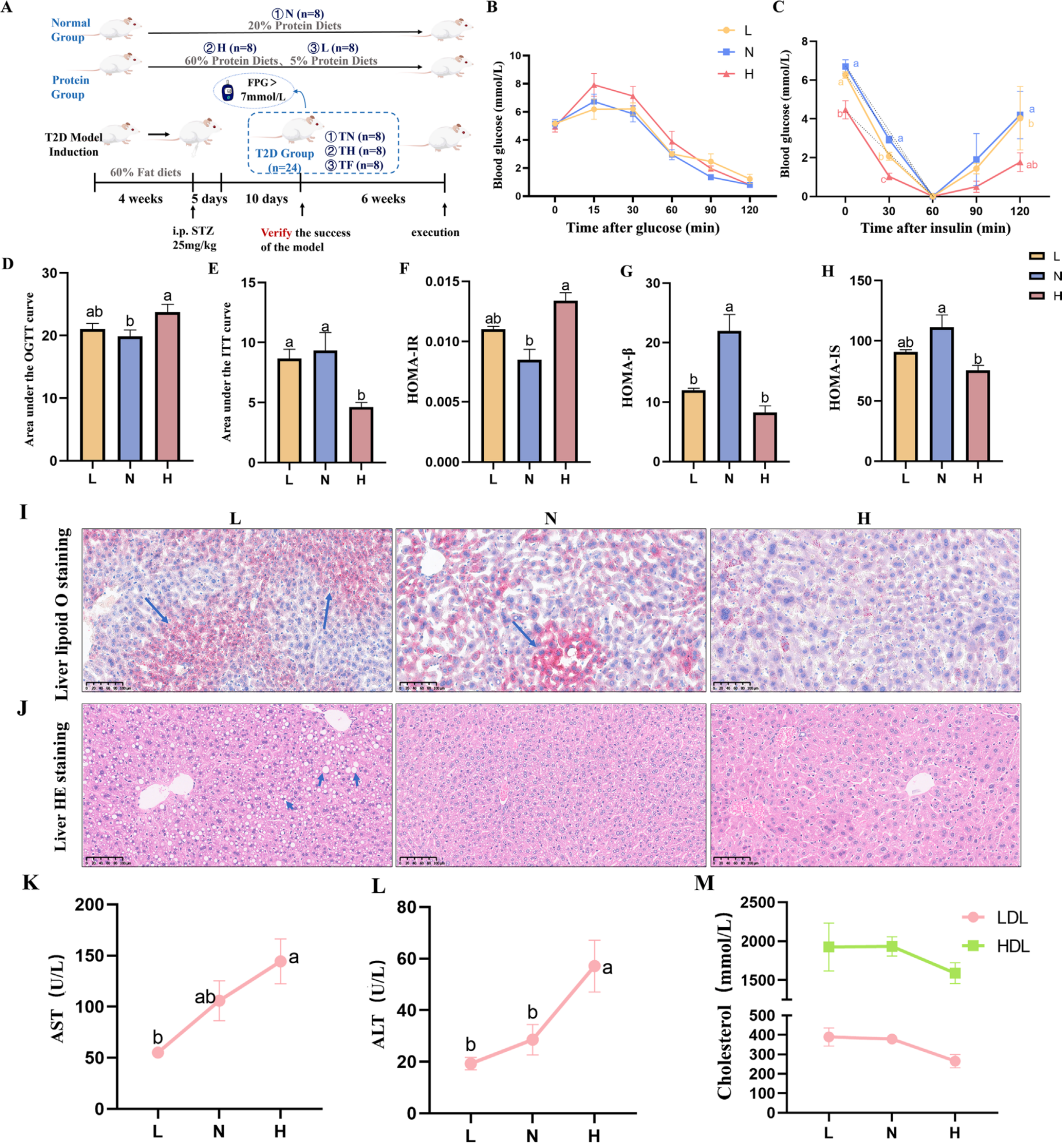
HP diet induces insulin resistance, impaired glucose tolerance, and alters liver function with lipid metabolism disorders in mice. (A) Experimental workflow diagram. (B) Glucose change curve during OGTT. (C) Glucose curve during ITT (baseline corrected at 60 min). (D) Area under the OGTT curve calculated from Figure B. (E) Area under the ITT curve calculated from Figure C. (F) HOMA‐IR = (fasting glucose×fasting insulin)/22.5. (G) HOMA‐β = 20 × fasting insulin/(fasting glucose‐3.5). (H) HOMA‐IS = (1/HOMA‐IR). (I) Representative images of liver tissue stained with Oil Red O (scale bar: 100 μm). (J) Representative images of liver tissue stained with HE (scale bar: 100 μm). (K) Serum AST levels. (L) Serum ALT levels. (M) Serum LDL and HDL levels. Data are expressed as mean ± SEM (*n* = 8). Different letters (a, b, c, d) indicate statistically significant differences between groups (*p* < 0.05), while identical letters denote no significant difference.

Five‐week‐old male KM mice were assigned to three distinct dietary groups: normal control (N), high‐protein diets (H), and low‐protein (L) diets groups, maintained for 12 weeks. The experimental workflow is depicted in Figure 1A. To assess glucose homeostasis regulation capacity [20], an OGTT was conducted after four weeks of dietary intervention. This test evaluates the rapid secretory capacity of pancreatic β‐cells and peripheral tissue glucose uptake efficiency following glucose challenge. We observed significantly higher peak blood glucose levels at 15 min in the H group compared to L and N groups, accompanied by delayed glucose clearance (Figure 1B). The area under the glucose curve (AUC) was markedly increased (*p* < 0.05; Figure 1D), indicating marked impaired glucose tolerance following glucose loading. One week later, an ITT test was performed by administering exogenous insulin to assess tissue responsiveness to insulin and the efficacy of glycogenolysis and gluconeogenesis [21]. The H group exhibited the lowest baseline blood glucose but the slowest decline (Figure 1C), along with reduced AUC (*p* < 0.05; Figure 1E), suggesting the HP diet impaired insulin sensitivity. Additionally, we assessed Homeostasis model assessment (HOMA) indices—calculated from fasting glucose and insulin levels—as core tools for evaluating diabetic pathophysiology [22]. The H group exhibited significantly elevated HOMA‐Insulin Resistance (HOMA‐IR) (*p* < 0.05; Figure 1F), directly demonstrating that the HP diet induced insulin resistance. HOMA‐Insulin sensitivity index (HOMA‐IS) was markedly decreased (*p* < 0.05; Figure 1H), further corroborating reduced insulin sensitivity. HOMA‐β‐cell function (HOMA‐β) (*p* < 0.05; Figure 1G) was significantly reduced, indicating impaired β‐cell function in HP‐fed mice. Collectively, the HP diet caused a dramatic decline in insulin‐mediated glucose‐lowering efficacy, ultimately triggering and exacerbating severe glycemic dysregulation.

### 3.2 HP Diet Alters Liver Function and Promotes Lipid Metabolic Disorders

Oil Red O staining specifically visualises neutral lipid (primarily TG) deposition in the liver, where hepatic fat accumulation is a key feature and driver of hepatic insulin resistance [23]. Excess lipids and their metabolites (e.g., ceramides, diacylglycerols) disrupt insulin signalling pathways within hepatocytes, leading to uninhibited hepatic glucose output and exacerbating fasting hyperglycemia [24]. Experimental results showed significantly fewer lipid droplets in the H group compared to the L group, which exhibited marked steatosis (Figure 1I). HE staining reveals the liver's overall tissue architecture, cellular morphology, and presence of inflammation or injury. Combined with Oil Red O staining, it translates abstract metabolic abnormalities (e.g., insulin resistance, inflammation) into visible pathological changes. This study found that hepatocytes in the H group exhibited intact structure and reduced lipid deposition (Figure 1J). AST and ALT serve as direct markers of hepatocyte injury. Serum AST and ALT levels were significantly elevated in Group H (Figure 1K,L). Concurrently, LDL and HDL levels—which influence complication development in T2D patients [25]—were reduced (Figure 1M), indicating persistent liver injury and lipid metabolism disorders despite diminished steatosis. The HP diet not only exacerbated hyperglycaemia and insulin resistance but also significantly increased the risk of complications in mice.

### 3.3 HP Diet Exacerbates Glucose Homeostasis in T2D Mice and Induces Insulin Resistance, Worsening Pancreatic β‐Cell Dysfunction

**FIGURE 2:**
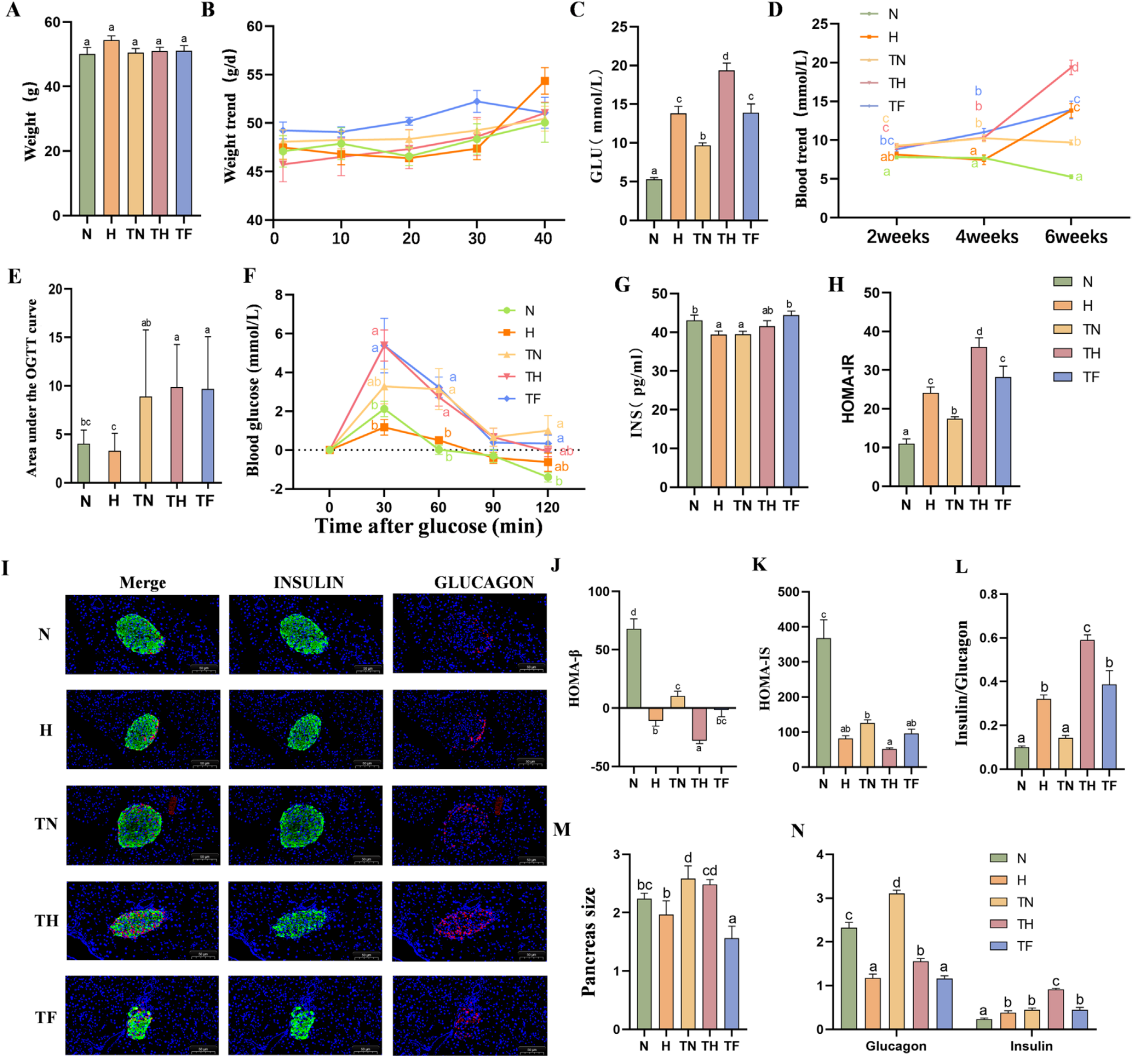
HP diet exacerbates glycemic homeostasis, induces insulin resistance, and impairs pancreatic β‐cell function in T2D mice. (A) Body weight of mice in each group at the end of intervention. (B) Changes in body weight during intervention. (C) Intergroup comparison of fasting blood glucose at week 6. (D) Changes in blood glucose levels during intervention. (E) AUC of OGTT. (F) Glucose change curves during OGTT in each group. (G) INS. (H) HOMA‐IR. (I) Representative images of pancreatic tissue insulin/glucagon immunofluorescence double staining (20×). (J) HOMA‐β. (K) HOMA‐IS. (L) Quantitative analysis of α/β cell ratio. (M) Pancreatic area size. (N) Quantitative analysis of α and β cell area proportions. Data are expressed as mean ± SEM (*n* = 8). Different letters (a, b, c, d) indicate statistically significant differences between groups (*p* < 0.05).

This study successfully established a T2D mouse model while setting up different protein diet groups. Specifically, mice were first fed a high‐fat diet for four weeks, followed by five consecutive days of STZ injections. Ten days later, blood glucose levels > 7 mmol/L confirmed successful modelling. All mice in this study met this criterion, achieving a 100% success rate. The successfully induced T2D mice were further divided into three groups: Normal Control (TN), High Protein (HP) Model (TH), and High Fat Model (TF) groups, each undergoing corresponding dietary interventions for 6 weeks. During the 6‐week intervention period, no significant differences in body weight were observed among the groups (Figure 2A). To continuously monitor model stability, fasting blood glucose was measured biweekly, revealing that T2D model mice consistently maintained elevated glucose levels. Notably, both the TH and TF groups exhibited a sustained upward trend in blood glucose levels (Figure 2D), with TH group mice showing significantly higher glucose levels than the diabetic control group by week 6 (Figure 2C). To further evaluate glucose tolerance in T2D mice, an OGTT was conducted. Results showed that TH group mice exhibited a sharp rise in blood glucose levels at 30 min post‐OGTT, comparable in magnitude to the TF group, followed by a decline; whereas the TF group maintained persistently elevated blood glucose levels (Figure 2F), indicating differences in glucose load processing capacity between the two groups. Both the H and TH groups exhibited significantly elevated HOMA‐IR indices (*p* < 0.05; Figure 2H), with the H group demonstrating insulin resistance exceeding that of the TN group, indicating that the high‐protein diet was associated with increased insulin resistance, as evidenced by elevated HOMA‐IR and OGTT results. Furthermore, the HOMA‐IS and HOMA‐β indices were significantly reduced in both the H and TH groups (*p* < 0.05; Figure 2J,K), reflecting that the HP diet impaired insulin sensitivity and β‐cell function in both normal and T2D mice. β‐cells are the sole source of insulin secretion, and their number and total volume form the material basis for maintaining normal insulin secretion capacity [26]. Pancreatic immunofluorescence double staining revealed a significant reduction in the proportion of pancreatic α‐cells in H, TH, and TF group mice, with a significantly increased α/β‐cell ratio in H and TH groups (*p* < 0.05; Figure 2L,N), and reduced mean islet size (*p* < 0.05; Figure 2M). These findings indicate impaired β‐cell function and disrupted endocrine architecture within the islets, potentially linked to high‐protein diet‐induced metabolic inflammation and insulin resistance.

### 3.4 HP Diet Induces Hepatic Steatosis and Functional Impairment Through Pro‐Inflammatory and Lipid Metabolism Dysregulation

**FIGURE 3:**
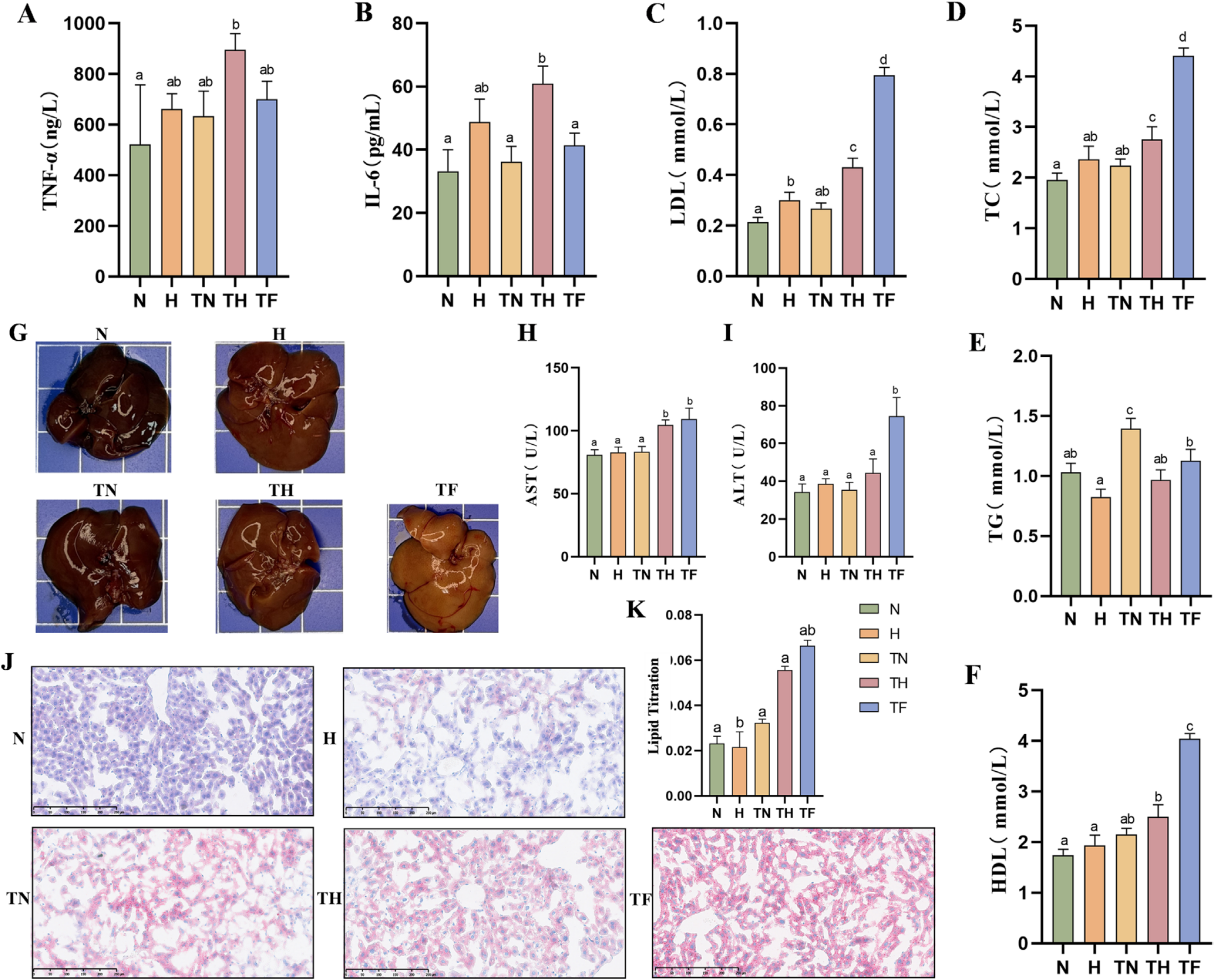
HP diet elevates inflammatory factors, disrupts lipid metabolism, and induces hepatic steatosis and functional impairment. (A) Liver TNF‐α levels. (B) Liver IL‐6 levels. (C) Serum LDL levels. (D) Serum TC levels. (E) TG levels. (F) HDL levels. (G) Representative histological appearance of mouse liver at the end of the experiment. (H) Serum AST levels. (I) Serum ALT levels. (J) Representative images of liver tissue stained with Oil Red O (scale bar: 100 μm). (K) Quantitative analysis of lipid droplet area by Oil Red O staining. Data are expressed as mean ± SEM (*n* = 8). Different letters (a, b, c, d) indicate statistically significant differences between groups (*p* < 0.05).

T2D similarly represents a chronic low‐grade inflammatory state, with TNF‐α and IL‐6 potentially driving worsening insulin resistance and β‐cell dysfunction [27]. We observed significantly elevated serum TNF‐α and IL‐6 levels in TH group mice compared to other groups (*p* < 0.05; Figure 3A,B). The H group exhibited a similar trend, indicating that HP intake itself possesses potent pro‐inflammatory effects. Regarding lipid metabolism, TC was significantly elevated in the H and TH groups, with the highest levels observed in the TF group (Figure 3D). Unexpectedly, neutral fat triglyceride (TG) levels increased in the N group (Figure 3E), while LDL was lowest in the N group (Figure 3C). HDL levels showed an upward trend across all groups (Figure 3F). Further examination of hepatic lipid deposition in T2D mice revealed the most severe hepatic steatosis in the TF group, characterised by pale liver coloration and elevated AST and ALT levels (Figure 3G–I). Oil Red O staining further confirmed significant lipid accumulation in the TH group (Figure 3J,K), indicating that HP intake exacerbates hepatic steatosis and impairs liver function in T2D mice.

### 3.5 HP Diet Activates IKKβ and JNK Signalling Pathways and Inhibits IRS‐1 Phosphorylation

**FIGURE 4:**
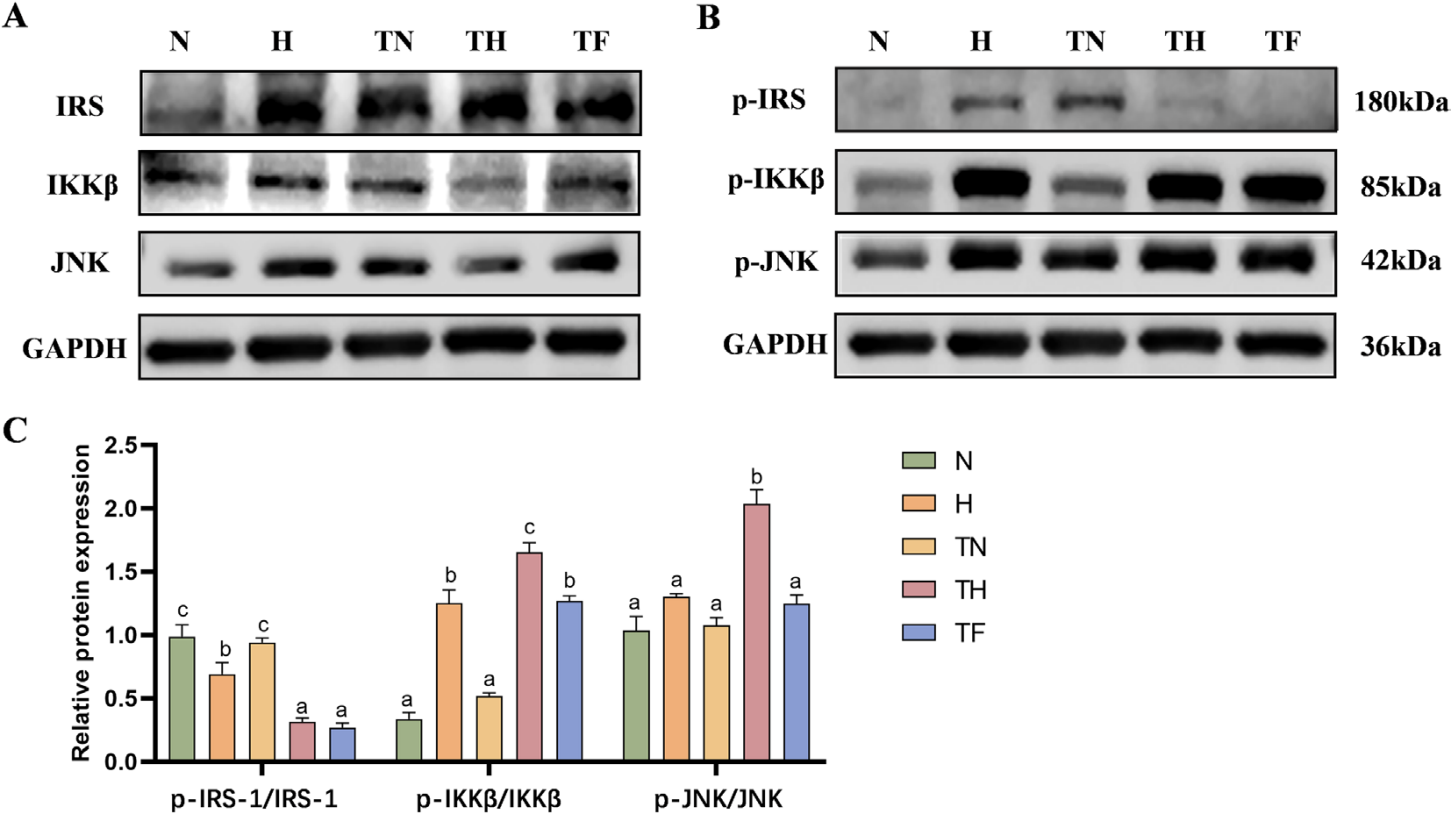
HP diet activates IKKβ and JNK signalling pathways and inhibits IRS‐1 phosphorylation. (A) Representative Western Blot bands showing IRS‐1, IKKβ, and JNK protein expression in liver tissue. (B) Representative Western Blot bands showing p‐IRS‐1, p‐IKKβ, and p‐JNK protein expression in liver tissue. (C) Relative quantitative analysis of p‐IRS‐1/IRS‐1, p‐IKKβ/IKKβ, and p‐JNK/JNK. Data are expressed as mean ± SEM (*n* = 3). Different letters (a, b, c, d) indicate statistically significant differences between groups (*p* < 0.05).

JNK and IKKβ play pivotal roles in the progression of diabetes. Both can modify key molecules in the insulin signalling pathway through specific phosphorylation events. They may also induce β‐cell apoptosis, disrupt normal insulin signalling transmission, thereby reducing insulin sensitivity, promoting the development of insulin resistance, and disrupting glucose homeostasis regulation [28, 29]. Western blot analysis revealed significantly reduced IRS‐1 phosphorylation levels in the H and TH groups compared to the N and TN groups (*p* < 0.05; Figure 4A–C), indicating impaired insulin signalling. Both H and TH groups exhibited enhanced IKKβ phosphorylation, while JNK phosphorylation increased significantly only in the TH group, suggesting differential activation of inflammatory signalling pathways between the HP and high‐fat diets.

## 4 Discussion

Since the introduction of the Atkins diet in the 1960s [30], the impact of protein intake on metabolic health has remained a subject of debate [31]. Although high‐protein diets are frequently recommended for weight management and body composition improvement [1, 32], their long‐term effects on glucose homeostasis and insulin sensitivity remain unclear [6]. Therefore, this study aimed to investigate the effects of an HP diet on insulin sensitivity in mice.

Glucose tolerance, insulin resistance coefficient, and pancreatic β‐cell function assessment are commonly used indicators for evaluating glucose homeostasis [33]. This study demonstrates that HP intake impairs glucose homeostasis in mice, manifested as reduced glucose disposal capacity, severely impaired insulin‐mediated blood glucose regulation, and compromised β‐cell insulin secretion. Previous studies indicate that protein intake enhances insulin resistance in non‐diabetic populations [1] and complicates glucose homeostasis [5], consistent with our findings. Glucose tolerance tests can identify abnormal glucose metabolism in mice [34]. We observed impaired glucose metabolism and worsened glucose tolerance in both normal and T2D mice fed the HP diet, aligning with prior research [35]. Insulin resistance occurs when pancreatic function cannot compensate for insulin resistance defects, leading to elevated blood glucose and progressive diabetes development [36]. HOMA‐IR values were elevated in both TH and TF groups, indicating impaired insulin sensitivity in T2D mice under both dietary conditions. Excessive protein intake may induce insulin resistance and T2D more readily than a high‐fat diet, similar to previous studies [1]. Pancreatic tissue size serves as a crucial morphological indicator for assessing islet β‐cell reserve and metabolic status [37]. Our experiments revealed varying degrees of islet atrophy in the High, H, and T2D model groups, accompanied by a significant reduction in β‐cell numbers. This structural alteration may be associated with β‐cell dedifferentiation and apoptosis, leading to partial loss of insulin secretion function [38]. Pancreatic atrophy is widely recognised as a hallmark pathological feature in the onset and progression of T2D [39], often indicating irreversible damage to β‐cell function. In summary, this study further confirms at both morphological and functional levels that HP diets exacerbate the pathological progression of T2D.

As a core organ for insulin signalling and glucose‐lipid metabolism regulation [13], liver health directly determines systemic glucose homeostasis [40]. HE staining in L group mice revealed hepatocyte swelling, cytoplasmic vacuolation, and clearly visible lipid vacuoles. Oil Red O staining visually demonstrated a significant increase in the proportion of neutral fats stained bright red. Additionally, T2D model mice exhibited increased hepatic lipid accumulation, manifested as yellowish liver coloration and greasy texture, whereas both H and TH groups showed reduced steatosis, consistent with previous studies [3]. However, the elevation of AST and ALT—sensitive markers of hepatocyte injury—in the TH group, coupled with reduced LDL and HDL levels, suggests that the HP diet exerts dual effects on the liver. While histological improvements occurred, potential risks of dyslipidemia and hepatocyte damage may persist.

This study further explored the inflammatory mechanisms by which the HP diet influences insulin sensitivity. TNF‐α, a key pro‐inflammatory cytokine, has been demonstrated to disrupt insulin signalling pathways by inhibiting tyrosine phosphorylation of IRS‐1 [41]. Additionally, studies have reported [13] that TNF‐α gene knockout improves insulin sensitivity in obese mice, suggesting its central role in metabolic disorders. In this study, we observed significantly elevated TNF‐α and IL‐6 levels in the HP diet groups (H and TH) with impaired glucose homeostasis, indicating systemic inflammation activation that may contribute to worsening insulin sensitivity [42].

At the molecular level, the HP diet may activate downstream JNK and IKKβ signalling pathways by upregulating TNF‐α and IL‐6. Previous studies have demonstrated that [43] cytokines such as TNF‐α and IL‐6 can activate pro‐inflammatory kinases (e.g., JNK and IKKβ), consistent with our findings. Experimental data revealed that HP intervention significantly enhanced the phosphorylation levels of IKKβ and JNK, accompanied by suppression of IRS‐1 activity. As a stress‐sensitive kinase, JNK can be activated by inflammatory factors and disrupts normal insulin signalling by directly phosphorylating serine sites on IRS‐1 [11, 44]. Similarly, IKKβ not only serves as a classic activator of the NF‐κB pathway but also phosphorylates IRS‐1, thereby interrupting downstream insulin signalling [9, 12]. Notably, IKKβ plays a central role in mediating insulin resistance, serving as a pivotal link between inflammation and dysregulation of glucose and lipid metabolism [13]. Insulin resistance frequently arises from modifications and functional abnormalities in insulin receptors and downstream signalling molecules such as IRS‐1 [14].

In summary, HP diets significantly impair pancreatic function and exacerbate insulin resistance, particularly in T2D mice. This mechanism is associated with β‐cell depletion, suppression of IRS‐1 activity, and inflammation, alongside activation of the IKKβ/JNK signalling pathway, accompanied by improved hepatic lipid accumulation but impaired liver function. While our data show a correlation between JNK/IKKβ activation and reduced β‐cell mass, direct causality cannot be concluded from this study. Our findings suggest that excessive protein intake induces pancreatic dysfunction and glucose metabolism disorders, offering new insights for dietary interventions and metabolic disease prevention.

## Author Contributions

All the authors contributed to the study conception and design. X.Y.: Conceptual design, methodology, and review. L.W.: Conceptual design and methodology. J.L.: Experimental operations, data organisation, writing, and data collection. J.X.: Experimental operations and data organisation. J.T.: Data organisation and data collection. L.C.: Experimental operations and data organisation. L.Z.: Experimental operations and data organisation. Y.Y.: Experimental operations and animal feeding. J.W.: Experimental procedures, animal feeding. L.Y.: Experimental procedures, animal feeding. Y.Y.: Experimental procedures, animal feeding. Y.J.: Experimental procedures. S.C.: Experimental procedures. B.W.: Experimental procedures. The initial draft was written by J.L., with all authors contributing revisions to earlier versions. All authors reviewed and approved the final manuscript.

## Funding

This work was supported by the Shanxi Provincial Key Research Laboratory Construction Project of Traditional Chinese Medicine (#zyyyyjs2024018) and the Shanxi Provincial Scientific and Technological Innovation Talent Team Special Project (#202204051001033).

## Ethics Statement

This study was conducted in accordance with the principles of the Declaration of Helsinki and approved by the Animal Ethics Committee of Shanxi Cancer Hospital (IACUC No.: 2024017). Experimental animals were mice from the Animal Center of Shanxi Cancer Hospital, housed in the SPF laboratory of the Animal Center. The experimental procedures strictly adhered to the ‘3R’ principle.

## Consent

The authors confirmed that human research participants provided informed consent for all images in the release chart.

## Conflicts of Interest

The authors declare no conflicts of interest.

## Data Availability

The datasets generated during and/or analysed during the current study are available from the corresponding author upon reasonable request.
